# Effects of Electron Radiation on Serotonin Signaling and Reactivity of Rat Gastric Smooth Muscle

**DOI:** 10.3390/toxics11070603

**Published:** 2023-07-12

**Authors:** Raina Ardasheva, Natalia Prissadova, Valentin Turiyski, Anna Tolekova, Athanas Krastev, Mina Pencheva, Veselin Popov

**Affiliations:** 1 Department of Medical Physics and Biophysics, Faculty of Pharmacy, Medical University of Plovdiv, 4002 Plovdiv, Bulgaria; 2 Medical College, Trakia University, 6015 Stara Zagora, Bulgaria; 3 Department of Clinical Oncology (Section of Radiotherapy and Nuclear Medicine), Faculty of Medicine, Medical University of Plovdiv, 4002 Plovdiv, Bulgaria

**Keywords:** accelerated electron beam, serotonin, 5-HT receptors, gastric smooth muscle

## Abstract

Ionizing radiation in radiotherapy can disrupt cellular functions based on radiation type, energy, and dose. However, investigations on the effects of accelerated electrons, particularly on serotonin mediation, are limited. This study aimed to investigate changes in serotonin signal transduction (targeting 5-HT_2A_ and 5-HT_2B_ receptors) in gastric smooth muscle (SM) samples isolated from rats irradiated with accelerated electrons (linear accelerator Siemens Primus S/N 3561) and their effects on serotonin-induced reactions. The radiation effects were examined in samples prepared five days after the procedure. The contractile activity of smooth muscle samples was measured using an isometric method. The expression of 5-HT_2A_ and 5-HT_2B_ receptors was determined by immunohistochemical assay. Increased contractile reactivity to exogenous serotonin (1.10^−8^–1.10^−4^ mol/L) was observed in irradiated samples compared to controls. The expression of 5-HT_2A_ and 5-HT_2B_ receptors was significantly increased in the irradiated tissue. By selecting appropriate time intervals between equimolar (1.10^−6^ mol/L) sequential serotonin exposures, a process of desensitization associated with agonist-induced internalization was established in control samples, which was absent in irradiated samples. In conclusion, irradiation with accelerated electrons affects the agonist-induced receptor internalization of 5-HT_2A_ and 5-HT_2B_ receptors and increases their expression in rat gastric SM, which alters their contractile reactivity to exogenous serotonin.

## 1. Introduction

Radiation therapy is used in patients with various types of cancer, as well as for the treatment of some benign tumors. The ionizing radiation used, depending on the type of radiation, energy, and absorbed dose, is a factor that causes disturbances in many cellular functions [1] and affects the nature and strength of various processes at the cellular, tissue, and organism levels. The causes of such disturbances at the cellular level are usually a combination of changes related to disrupted synthesis and release of neurotransmitters, activation/inhibition of enzyme systems and intracellular signaling pathways, influence on receptors, modifications of biomolecules, unlocking mechanisms regulating the expression or synthesis of cellular structures, etc. [2]. Despite increasing knowledge on the cellular and molecular mechanisms of radiation damage, relatively little is known about the specific effects of electron radiation, which is used in some cases as a method in radiation therapy; in particular, almost nothing is known about its effect on serotonin neurotransmission. Serotonin [5-hydroxytryptamine (5-HT)] is a multifunctional neurotransmitter that mediates a number of vital neuronal, somatic, and behavioral processes in the body [3,4]. Serotonin-induced effects are mediated by a family of 5-HT receptors, which (excluding 5-HT_3_) belong to the group of G protein-coupled receptors (GPCRs). A common characteristic is their involvement in a system cyclic process of desensitization (including internalization, resensitization, and recycling to the plasma membrane), induced by the corresponding agonists [5], which affects the GPCR signaling. It is believed that the proper coordination of these events usually reduces its strength [6] and protects cells from excessive receptor stimulation and the corresponding reaction [7]. A large part of the processes provoked by serotonin is the result of the direct participation of 5-HT_2A_ and 5-HT_2B_ receptors, activating a diverse set of intracellular pathways [8].

A major concern is the impact of ionizing radiation on serotonin mediation and its functions. The whole-body irradiation of mice (0.5 Gy, 1 Gy and 3 Gy ionizing radiation) provokes alteration in normal behavior over 10 days. Serotonin levels have undergone changes in the blood, hippocampus, and whole brain tissue [9], and cognitive impairment has been observed. Kokhan et al. reported an effect on the protein content of 5-HT_2A_ and 5-HT_4_ receptors in the prefrontal cortex, as well as a decreased content of serotonin transporter and increased content of tryptophan hydroxylase in the hypothalamus of irradiated rats [10]. Exposure to ionizing radiation leads to changes in psycho-emotional and neuroadaptive states, related to 5-HT (predominantly through 5-HT_2C_, 5-HT_3_ and 5-HT_4_ receptors) innervation in stressful conditions such as cosmic irradiation [11]. Other scientists have found that ionizing electromagnetic radiation alters some of the GI tract function through 5-HT modulated pathways [12].

Electron radiation has specific physical mechanisms of penetration and interaction with matter, different from those of photon radiation. Accelerated electron beams penetrate at different depths into tissues and phantoms, depending on their energy [13]. Even though most electron energy is absorbed superficially, electron beams can treat tumors at a 5 cm depth [14] and even up to a depth of 4 inches [15]. Entering the body, accelerated electrons provoke ionizing-induced effects on target tissues, and these effects could cause functional and/or structural changes in the 5-HT receptor [16,17]. However, the effect of electron radiation on serotonin signaling and the resulting effects have not been studied well enough. Further exploration of the area may provide a new perspective on the mechanisms of radiation-induced disturbances in the body.

The aim of the present study was to investigate changes in serotonin signal transduction (targeting 5-HT_2A_ and 5-HT_2B_ receptors) in gastric smooth muscles (SMs) isolated from rats irradiated with accelerated electrons and their effects on serotonin-induced reactions.

## 2. Materials and Methods

### 2.1. Animals

Smooth muscle samples from a gastric corpus of rats were used for the purpose of our study. The reason for that is the high number of 5-HT receptors in SM tissue of the gastro-intestinal (GI) tract [18,19].

Male Wistar rats with a body weight in the range of 250–280 g were provided by the Animal house of Medical University—Plovdiv, Bulgaria. Rats were bred in standard laboratory conditions (23–25 °C, 50–55% humidity and 12/12 h light/dark cycle). They had ad libitum access to food and water and were deprived of food for 24 h before euthanasia.

All experiments were carried out according to the European Union (directive 2010/63/EU) and Bulgarian guidelines (directive No. 20/01.11.2012) for using laboratory animals (License No. 213/5.10.2018 from the Animal Health and Welfare Directorate of the Bulgarian food safety agency (BFSA, https://bfsa.egov.bg/wps/portal/bfsa-web-en/home (accessed on 11 July 2023))).

### 2.2. Irradiation Procedures

The anatomical topographic planning involved choosing an optimal rat position that would provide repeatability and reproducibility in subsequent irradiations. The rats were anesthetized in advance (combination of xylazine 2%–10 mg/kg + ketamine (calipsol) 5%–100 mg/kg, administered as an intraperitoneal injection) and immobilized in a dorsal position on a plexiglass mat matching their size (30 × 30 × 0.5 cm). The planning was performed using a Siemens Somatom Spirit Power (Siemens, Munich, Germany) computed tomograph, the Abdomen Routine Radiotherapy protocol and spiral scanning with parameters of 100 kV and 120 mAs. A sequence of 80 consecutive transverse tissue sections was obtained, each one being 3 mm thick. The rat’s outline was delineated in each transverse section obtained by the computed tomography investigation. The CMS XiO treatment planning system was used for that purpose, as well as for the three-dimensional dosimetric planning that followed.

The experimental animals received whole-body irradiation. The energy of the electron beam was 9 MeV. A standard symmetrical electron applicator was utilized—25 × 25 cm. The planned single irradiation dose—5 Gy—was determined at the depth of the dosage maximum of the energy used (2 cm). The procedures were carried out using a Siemens Primus S/N3561 (Siemens, Munich, Germany) multimodality linear accelerator.

The mathematical algorithm used to calculate the received dose was Pencil Beam. Before the irradiation, the rats were centered abdominally, which provided identical body positions in both planning and irradiating. After completing the session, the rats were transported to a laboratory for further investigation.

### 2.3. In Vitro Experiments

At the beginning of the in vitro experiments, animals were euthanized by overdose anesthesia (5 times the doses of Ketamin (100 mg/kg) and Xylazin (10 mg/kg)).

#### 2.3.1. Isometrical Registration of SM Contractile Activity

SM preparations from the circular muscle layer of a stomach-corpus were cut. They had a width of 1.0–1.1 mm and a length of 13–15 mm. The contractile activity of the preparations was registered isometrically by Tenzo “Swema” detectors—Sweden. The initial mechanical stress of the preparations reached by the Tenzo stretch system corresponds to a tensile force of 10 mN. Krebs solution (pH = 7.4) used for washing SM preparations has been continuously aerated with a gas mixture of 95% O_2_ and 5% CO_2_ at 37 °C. The 60 min adaptation of the tone level of preparations was taken as a starting tone, and the changes as contraction or relaxation were compared to it. During the period of adaptation, Krebs solution in the tissue baths was replaced several times. The drug-caused reactivity of SM preparations was counted and registered by Microtechna gain stage (Microtechna, Prague, Czech Republic) and recorded on the Linseis paper recorder (Linseis, Selb, Germany).

Composition of the Krebs solution (mM): NaCl 120; KCl 5.9; CaCl_2_ 2.5; MgCl_2_ 1.2; NaH_2_PO_4_ 1.2; NaHCO_3_ 15.4; glucose 11.5.

#### 2.3.2. Immunohistochemistry

Immunohistochemical studies were performed on paraffin-embedded sections (4–5 µm) obtained from tissues of control and irradiated rats (*n* = 6), mounted on glass microscope slides and secured by means of an adhesive.

The sections obtained from the gastric wall of the rat were deparaffinized, then subjected to the following procedures: detection of antigenic epitopes with citrate buffer, blocking endogenous peroxidase with 3% hydrogen peroxide, blocking endogenous biotin using a kit (ref: No. BBK 120, Scy Tek, Lab., Inc., Logan, UT, USA), blocking non-specific binding using a reagent (Superblock, Scy Tek, Lab., Inc., Logan, UT, USA), followed by incubation for 24 h (at 4 °C) with specific mouse monoclonal antibody against SR-2A (A-4): sc-166775 or SR-2B (C-6): sc-376878 (Santa Cruz Biot., CA, USA, 1:300 solution), after which a second 10 min incubation followed, with a biotinylated secondary antibody (No. AGL015 Scy Tek Lab., Inc., Logan, UT, USA). The reaction was visualized by 3,3′-diaminobenzidine tetrachloride (DAB, Scy Tek Lab., Inc., Logan, UT, USA), and the slices were counterstained with Mayer’s hematoxylin.

All microphotographs were taken using a Nikon Microphot SA (Nikon, Tokyo, Japan) microscope combined with a Camedia-5050Z digital camera (Olympus, Tokyo, Japan). The preparations were observed at a magnification ×400.

#### 2.3.3. Morphometric Analysis of Immunoreactions for 5-HT_2A_ and 5-HT_2B_

The morphometric analysis involved tissue slices 4 μm in thickness, obtained from a rat stomach. The intensity of the immune reaction in the smooth muscle cells was measured in arbitrary units (AU) on the slices immunostained for 5-HT_2A_ and 5-HT_2B_ receptors. Using software, the average intensity of pixels was recorded in arbitrary units in the range of 0–256 on microphotographs of the smooth muscle cells, 0 being black and 256 being white. A minimum of 50 points were measured in the smooth muscle cells, each slice at magnification ×400. All measurements involved five slices per animal. The measurements were performed using the DP–Soft ver. 3.2 software, Olympus, Tokyo, Japan.

#### 2.3.4. Drugs and Chemicals

The following drugs and chemicals were used: Serotonin (5-HT) (Sigma-Aldrich, Merck, Saint Louis, MO, USA), Acetylcholine (Sigma-Aldrich, Merck, Saint Louis, MO, USA), mouse monoclonal antibodies SR-2A (A-4): sc-166775 or SR-2B (C-6): sc-376878 (Santa Cruz Biot., CA, USA), buffer for rinsing (50 mM TRIS, pH 7.6, 150 mM NaCl2, 0.05% Tween-20, TTBS), Biotin Blocking Kit, cat. No. BBK 120 (ScyTek Lab., Inc., Logan, UT USA); 3,3′-diaminobenzidine tetrahydrochlorid (DAB, cat. No. ACV500, Scy Tek, Lab., Inc., Logan, UT, USA), alcohol solutions (70%, 80%, 96%, 100%), xylene. All the ingredients for Krebs solution (NaCl, KCl, CaCl_2_, MgCl_2_, NaH_2_PO_4_, NaHCO_3_ and glucose) were obtained from Merck (Darmstadt, Germany).

### 2.4. Statistical Analyses

The data obtained are expressed as the mean ± standard error of the mean (SEM). The number of preparations used in each experiment is indicated by n. Statistical differences were tested using Student’s *t*-test, and a probability (*p* < 0.05) was considered significant. All statistical analyses were performed using the specialized software SPSS, version 17.0 (SPSS Inc. Chicago, IL, USA).

## 3. Results

### 3.1. Mechanical Reactivity of Isolated SM to Increasing Concentration of 5-HT

**Figure 1:**
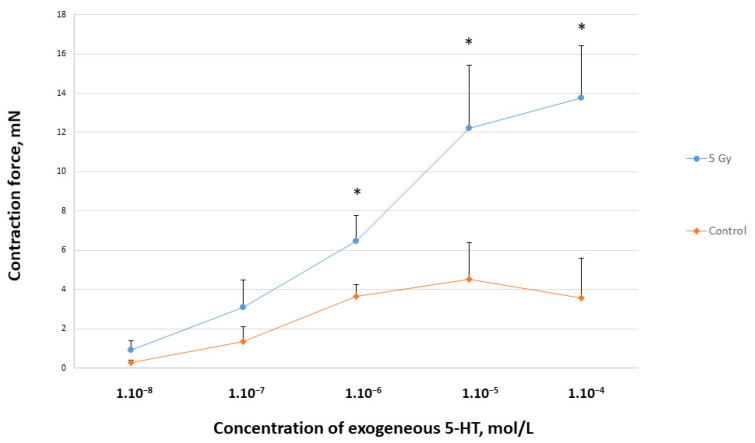
Concentration-effect curves of contractile reactivity to 5-HT of SM samples dissected from control and irradiated rats. *—comparison between reactions caused by equimolar concentrations of 5-HT in both types of tissues; *—*p* < 0.05.

We measured the smooth muscle reactivity caused by increasing serotonin concentrations (1.10–8 mol/L–1.10–4 mol/L). An increased serotonin gastric SM reactivity was found in the irradiated samples (*n* = 8) compared to the control ones (*n* = 10) (Figure 1). It is visible from the concentration-effect curves that there is a significant difference between 1.10–6 and 1.10–4 mol/L serotonin concentrations.

### 3.2. Light-Microscopic Immunohistochemical Analysis of 5-HT_2A_ and 5-HT_2B_ Receptors

**Figure 2:**
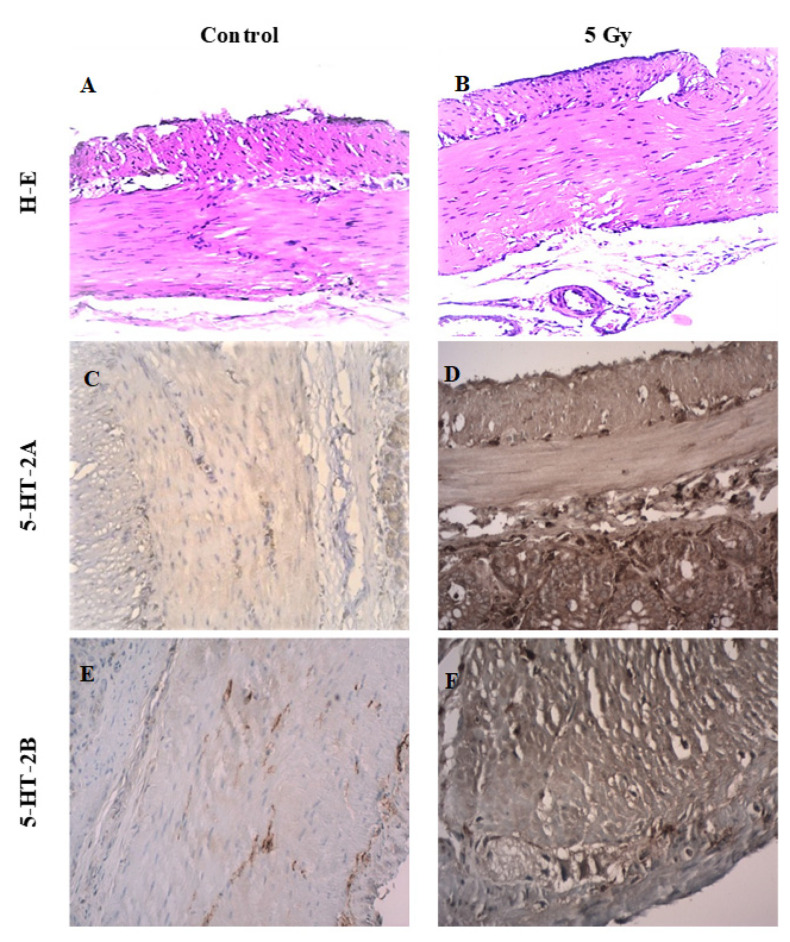
(**A**) Control SM samples stained with H-E; (**B**) irradiated SM samples stained with H-E; (**C**) immunohistochemical reaction of 5-HT_2A_ receptor in control SM samples; (**D**) immunohistochemical reaction of 5-HT_2A_ receptor in irradiated SM samples; (**E**) immunohistochemical reaction of 5-HT_2B_ receptor in control SM samples; (**F**) immunohistochemical reaction of 5-HT_2B_ receptor in irradiated SM samples; magnification for all—400×.

The immunohistochemical findings on the intensity of 5-HT_2A_ and 5-HT_2B_ receptors in SM of the stomach wall of irradiated and control rats, along with haematoxylin and eosin (H-E) staining, are shown in Figure 2. There are no visible morphological changes from the H-E staining between the groups (Figure 2A,B). We found an increased expression of the 5-HT_2A_ receptor in gastric SM cells from irradiated animals compared to the control (Figure 2C,D). Similarly, such an increase in the expression of the 5-HT_2B_ receptor was found in the irradiated samples (Figure 2E,F).

**Figure 3:**
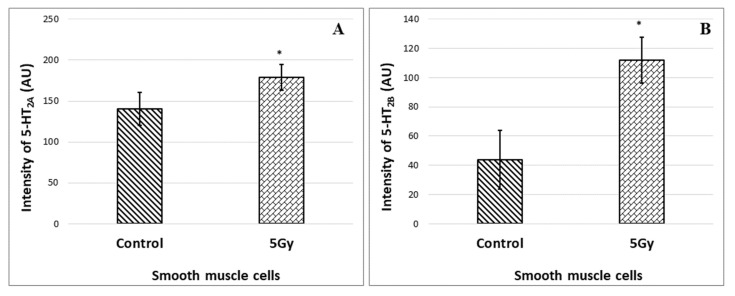
Quantitative representation of immunoreactivity for: (**A**) 5-HT_2A_ in the SM of the stomach wall; (**B**) 5-HT_2B_ receptors in the SM of the stomach wall. A comparison is made between preparations (*n* = 5) from the two groups of animals, separately for each type of receptor; *—*p* < 0.05.

The quantitative measurements of the 5-HT_2A_ and 5-HT_2B_ immunohistochemical reactions of the examined area are presented in Figure 3. The results confirm a statistically significant increase in the expression of both receptors in the irradiated samples.

### 3.3. Multiple Effects with Equimolar Concentrations (1.10^−6^ mol/L) of Serotonin

To find a possible explanation for the difference in the receptors’ expressions, we tested SM samples with equimolar concentrations of 5-HT. Table 1A shows the reactions of control samples (*n* = 9) treated four times consecutively with an equimolar concentration of 1.10^−6^ mol/L 5-HT, separated from each other by a 15–20 min pauses necessary for the recovery of the contractile activity. Data from a similar experiment, with a duration between the equimolar applications of more than 2.5 h, are presented in Table 1B.

The strength of the tonic reactions of irradiated SM samples caused by consecutive effects with an equimolar concentration of 1.10^−6^ mol/L 5-HT, with a pause of 15–20 min between every two consecutive applications, is illustrated in Table 2.

## 4. Discussion

Our study identified several specific features in the tissue reactions resulting from exposure to accelerated electrons. We chose a dose of 5 Gy due to the highest radiobiological impacts on serotonin-energetic mediation. The data for the last are based on our previous experiments with electron radiation (dose range from 1 to 8 Gy) [20] and similar experiments [21]. Contractile serotonin-induced (1.10^−8^–1.10^−4^ mol/L) responses in irradiated samples, at equivalent concentrations, were stronger compared to those in non-irradiated samples. It can be assumed that 5-HT_2A_ and 5-HT_2B_ receptors are mostly involved in SM responses in our experiments [19,22,23,24]. This provided a basis for us to investigate the effect of electron radiation on the agonist-induced response through immunohistochemical studies, while focusing on these receptors.

The results showed that 5 days after irradiation, the expression of 5-HT_2A_ and 5-HT_2B_ receptors significantly increased. This fact is evidence of the influence of electron radiation on processes affecting the number of plasma membrane receptors, causing increased expression in the irradiated samples. Based on the assumption that the amplitude of the response is proportional to the number of functionally active receptors and that one serotonin molecule combines with one receptor [25], we believe that the increased expression of 5-HT_2A_ and 5-HT_2B_ receptors explains the higher reactivity of irradiated samples.

Non-irradiated samples exhibit maximal contraction after exposure to 1.10^−5^ mol/L 5-HT. In our study, at higher concentrations of the substance (1.10^−4^ mol/L), the strength of the agonistic effect decreases. We attribute the reduced efficacy of serotonin in activating 5-HT receptors and the associated signal transduction above a certain concentration to agonist-induced internalization, which reduces the number of plasma membrane receptors. This is typical for GPCRs [6], which most 5-HT receptors are [26,27], and is a concentration-dependent and time-dependent process [28].

We hypothesize that the same process of desensitization is responsible for the reduction in the response strength of non-irradiated samples in experiments with sequential 4-fold equimolar interactions with 1.10^−6^ mol/L serotonin, separated by 15–20 min intervals. The mechanisms of GPCR receptor desensitization vary depending on the cellular and receptor type [25], with a prevailing reduction in the number of functioning receptors and affinity towards the agonist [29]. It is probable that the main reason for the reduction in the strength of the latest 5-HT-induced response is a decreased number of plasma 5-HT receptors, caused by their internalization from previous serotonin-induced interactions.

However, a similar process is absent in the control samples after increasing the duration between individual sequential interactions to 2.5 h. This difference can be explained by the fact that internalized 5-HT_2A_ receptors recycle back to the plasma membrane for about 2.5 h [5,30]. The experiments showed that the sequential responses do not differ significantly in strength. This suggests that the absence of serotonin interaction for such a period (2.5 h) largely provides receptor recycling and ensures a relatively constant membrane expression at the beginning of each subsequent interaction with 5-HT. In our opinion, the reason for the alterations in the reactions between the two groups is the different possibilities in the development and manifestation of agonist-provoked internalization processes of 5-HT_2A_ and 5-HT_2B_ receptors.

As noted, irradiated samples increase the steepness of serotonin-induced responses with an increase in agonist concentration in the range of 1.10^−8^–1.10^−4^ mol/L. The concentration-dependent delayed increase in the effect and its reversal at concentrations higher than 1.10^−5^ mol/L is present in non-irradiated tissues. Such effect is absent in the irradiated samples. This indicates the lack or strong inhibition of serotonin-induced desensitization, starting with the internalization of 5-HT receptors, and demonstrates the influence of electron radiation. Similarly, an increase in 5-HT_2A_ receptor signaling and the following reaction, due to inhibition of agonist-induced 5-HT_2A_ receptor internalization, was registered by Xia et al. [31].

The presence of such an influence on the internalization of 5-HT receptors explains the equally strong reactions of irradiated samples, unlike non-irradiated ones, during the 4-fold equimolar exposures with 1.10^−6^ mol/L 5-HT at intervals of 20 min. From this point of view, irradiation influences 5-HT receptor internalization and creates a relatively constant receptor expression—a prerequisite for equally significant serotonin-induced contractions.

Our thesis about the influence of electron radiation on the process of internalization–recycling of 5-HT_2A_ and 5-HT_2B_ receptors is supported by the lack of a reliable difference in the strength of four consecutive serotonin-induced responses of irradiated samples with an interval between exposures of 2.5 h.

## 5. Conclusions

Given the experimental design, the observed features of serotonin-induced reactivity in gastric SM samples are obviously solely a result of the influence of accelerated electron flow. This determines the effect of electronic radiation as a factor that, 5 days after irradiation:

- stimulates the expression of 5-HT_2A_ and 5-HT_2B_ receptors in SM cells from the gastric corpus;
- affects the agonist-induced internalization–recycling process of 5-HT_2A_ and 5-HT_2B_ receptors and, consequently, the cellular 5-HT signaling pathway in these tissues.

## Figures and Tables

**Table 1:** SM reactions induced by consecutive application of 1.10^−6^ mol/L 5-HT to preparations obtained from control rats with (A) 15–20 min recovery period between the applications and (B) 2.5 h recovery period between the applications. The strength of each of the reactions was compared with the initial one (treatment No. 1), separately for the two groups of preparations; *—*p* < 0.05.

No. Treatment	Force of Reactions, mN	
	A (*n* = 9)	B (*n* = 6)
1	3.75 ± 0.88	3.81 ± 0.82
2	3.63 ± 0.94 (*p* = 0.784; *t* = 0.278)	4.05 ± 0.66 (*p* = 0.599; *t* = 0.542)
3	3.03 ± 0.63 (*p* = 0.067; *t* = 1.963)	3.88 ± 0.89 (*p* = 0.891; *t* = 0.140)
4	2.82 ± 0.44 * (*p* = 0.014; *t* = 2.750)	3.81 ± 1.00 (*p* = 0.900; *t* = 0.082)

**Table 2:** Strength of consecutive contractions caused by 1.10^−6^ mol/L 5-HT in irradiated SM preparations with (A) 15–20 min recovery period between the applications and (B) 2.5 h recovery period between the applications. The strength of each of the reactions was compared with the initial one (treatment No. 1).

No. Treatment	Force of Reactions, mN	
	A (*n* = 8)	B (*n* = 9)
1	6.24 ± 0.85	6.03 ± 0.23
2	6.35 ± 0.82 (*p* = 0.798; *t* = 0.261)	5.74 ± 0.22 (*p* = 0.341; *t* = 0.98)
3	6.41 ± 0.79 (*p* = 0.681; *t* = 0.420)	5.81 ± 0.36 (*p* = 0.618; *t* = 0.51)
4	6.21 ± 0.77 (*p* = 0.940; *t* = 0.076)	5.68 ± 0.32 (*p* = 0.329; *t* = 1.01)

## Data Availability

All materials are presented in the manuscript.

## References

[1] Shadad A.K., Sullivan F.J., Martin J.D., Egan L.J. (2013). Gastrointestinal radiation injury: Symptoms, risk factors and mechanisms. World J. Gastroenterol..

[2] Reisz J.A., Bansal N., Qian J., Zhao W., Furdui C.M. (2014). Effects of Ionizing Radiation on Biological Molecules—Mechanisms of Damage and Emerging Methods of Detection. Antioxid. Redox. Signal..

[3] Lopez-Vilchez I., Diaz-Ricart M., White J.G., Escolar G., Galan A.M. (2009). Serotonin enhances platelet procoagulant properties and their activation induced during platelet tissue factor uptake. Cardiovasc. Res..

[4] Nautiyal K.M., Hen R. (2017). Serotonin receptors in depression: From A to B. F1000 Res..

[5] Raote I., Bhattacharyya S., Panicker M.M. (2013). Functional Selectivity in Serotonin Receptor 2A (5-HT2A) Endocytosis, Recycling, and Phosphorylation. Mol. Pharmacol..

[6] Ferguson S.S. (2001). Evolving concepts in G protein-coupled receptor endocytosis: The role in receptor desensitization and signaling. Pharmacol. Rev..

[7] Koppen C.J., Jakobs K.H. (2004). Arrestin-independent internalization of G protein-coupled receptors. Mol. Pharmacol..

[8] Mohammad-Zadeh L.F., Moses L., Gwaltney-Brant S.M. (2008). Serotonin: A review. J. Vet. Pharmacol. Therap..

[9] Bekal M., Sun L., Ueno S., Moritake T. (2021). Neurobehavioral effects of acute low-dose whole-body irradiation. J. Rad. Res..

[10] Kokhan V.S., Mariasina S., Pikalov V.A., Abaimov D.A., Somasundaram S.G., Kirkland C.E., Aliev G. (2022). Neurokinin-1 Receptor Antagonist Reverses Functional CNS Alteration Caused by Combined γ-rays and Carbon Nuclei Irradiation. CNS Neurol. Disord. Drug Targets.

[11] Kokhan V.S., Shakhbazian E.V., Markova N.A. (2019). Psycho-emotional status but not cognition is changed under the combined effect of ionizing radiations at doses related to deep space missions. Behav. Brain Res..

[12] Francois A., Ksas B., Gourmelon P., Griffiths N.M. (2000). Changes in 5-HT-mediated pathways in radiation-induced attenuation and recovery of ion transport in rat colon. Am. J. Physiol. Gastrointest. Liver Physiol.

[13] Ghorbani M., Tabatabaei Z.S., Noghreiyan A.V., Vosoughi H., Knaup C. (2015). Effect of Tissue Composition on Dose Distribution in Electron Beam Radiotherapy. J. Biomed. Phys. Eng..

[14] Das I.J., Kase K.R., Copeland J.F., Fitzgerald T.J. (1991). Electron beam modifications for the treatment of superficial malignancies. Int. J. Radiat. Oncol. Biol. Phys..

[15] Hasanpour S., Rahimi S., Makki O.F., Shahhosseini G., Khosravi A. (2016). Protective Influence of Gamma Rays and Electron-Beam Irradiation with a Commercial Toxin Binder on Toxic Effects of Aflatoxin B1 in Japanese Quails. Iran J. Toxicol..

[16] Curtis J., Vo T.K.N., Seymour C.B., Mothersill C.E. (2020). 5-HT2A and 5-HT3 receptors contribute to the exacerbation of targeted and non-targeted effects of ionizing radiation-induced cell death in human colon carcinoma cells. Int. J. Radiat. Biol..

[17] Cockerham L.G., Forcino C.D. (1995). Effect of Antihistamines, Disodium Cromoglycate (DSCG) or Methysergide on Post-irradiation Cerebral Blood Flowand Mean Systemic Arterial Blood Pressure in Primates after 25 Gy, Whole-body, Gamma Irradiation. J. Rad. Res..

[18] Komada T., Yano S. (2007). Pharmacological characterization of 5-Hydroxytryptamine-receptor subtypes in circular muscle from the rat stomach. Biol. Pharm. Bull..

[19] Hannon J., Hoyer D. (2008). Molecular biology of 5-HT receptors. Behav. Brain Res..

[20] Charilaos X.A., Ardasheva R.G., Popov V.G., Prissadova N.A., Turiyski V.I., Krustev A.D., Vlaykova M.I., Grudeva-Popova Z.G., Sopadzhieva M.V. (2019). Changes in the Contractile Activity and Reactivity to 5-HT of Smooth Muscles of Rats Following Total Body Irradiation with Accelerated Electrons. Folia Med..

[21] Wang S.W., Ren B.R., Qian F., Luo X.Z., Tang X., Peng X.C., Huang J.R., Tang F.R. (2019). Radioprotective effect of epimedium on neurogenesis and cognition after acute radiation exposure. Neurosci. Res..

[22] Borman R.A., Burleigh D.E. (1997). 5-HT1D and 5-HT2B receptors mediate contraction of smooth muscle in human small intestine. Ann. NY Acad. Sci..

[23] Janssen P., Prins N.H., Meulemans A.L., Lefebvre R.A. (2002). Smooth muscle 5-HT2A receptors mediating contraction of porcine isolated proximal stomach strips. Br. J. Pharmacol..

[24] Zhao A., Urban J.F., Morimoto M., Elfrey J.E., Madden K.B., Finkelman F.D., Shea-Donohue T. (2006). Contribution of 5-HT2A receptor in nematode infection-induced murine intestinal smooth muscle hypercontractility. Gastroenterology.

[25] Strange P.G. (2008). Agonist binding, agonist affinity and agonist efficacy at G protein-coupled receptors. Br. J. Pharmacol..

[26] Bohn L.M., Schmid C.L. (2010). Serotonin receptor signaling and regulation via β-arrestins. Crit. Rev. Biochem. Mol. Biol..

[27] McCorvy J.D., Roth B.L. (2015). Structure and Function of Serotonin G protein Coupled Receptors. Pharmacol. Ther..

[28] Levoye A., Zwier J.M., Jaracz-Ros A., Klipfel L., Cottet M., Maurel D., Bdioui S., Balabanian K., Prézeau L., Trinquet E. (2015). A Broad G Protein-Coupled Receptor Internalization Assay that Combines SNAP-Tag Labeling, Diffusion-Enhanced Resonance Energy Transfer, and a Highly Emissive Terbium Cryptate. Front. Endocrinol..

[29] Roth B.L. (2011). 5-HT2A serotonin receptor biology: Interacting proteins, kinases and paradoxical regulation. Neuropharmacology.

[30] Bhattacharyya S., Puri S., Miledi R., Panicker M.M. (2002). Internalization and recycling of 5-HT2A receptors activated by serotonin and protein kinase C-mediated mechanisms. PNAS.

[31] Xia Z., Gray J.A., Compton-Roth B.A., Roth B.L. (2003). A Direct Interaction of PSD-95 with 5-HT2A Serotonin Receptors Regulates Receptor Trafficking and Signal Transduction. JBC.

